# Delirium assessment in daily critical care with the CAM-ICU: a multicenter study

**DOI:** 10.1186/cc9756

**Published:** 2011-03-11

**Authors:** MM Van Eijk, M Van den Boogaard, AJ Slooter

**Affiliations:** 1 University Medical Center Utrecht, the Netherlands; 2 Radboud University Nijmegen Medical Center, Nijmegen, the Netherlands

## Introduction

Delirium occurs frequently in the ICU and is associated with poor outcome. Screening for delirium in ICU patients is recommended by several medical organizations to improve prognosis by early diagnosis and treatment. The Confusion Assessment Method for the ICU (CAM-ICU) has high sensitivity and specificity for delirium when administered by research nurses. However, the test characteristics of the CAM-ICU as performed in routine practice are unclear. The objective of this study is to investigate the diagnostic value of the CAM-ICU in daily practice.

## Methods

Teams of three alternating delirium experts including psychiatrists, geriatricians and neurologists visited 10 ICUs twice. Based on cognitive examination, inspection of medical files and DSM-IV-TR criteria for delirium, the expert teams classified patients as awake and not delirious, delirious or comatose. This classification served as the gold standard to which the CAM-ICU as performed by the bedside ICU nurses was compared. Assessors were unaware of each others' conclusions.

## Results

Thirteen delirium experts assessed 282 patients, of whom 101 (36%) were classified as comatose and excluded. In the remaining 181 (64%) patients, delirium was diagnosed in 75 by the experts of whom 35 scored CAM-ICU positive. This yielded a sensitivity of 47% (95% CI = 35 to 58%), specificity of 98% (95% CI = 93 to 100%), positive predictive value of 95% (95% CI = 80 to 99%) and negative predictive value of 72% (95% CI = 64 to 79%).

## Conclusions

Specificity of the CAM-ICU as performed in routine, daily practice appears to be high but sensitivity low. The low sensitivity hampers early detection of delirium by the CAM-ICU.

